# Low Temperature Dissolution of Yeast Chitin-Glucan Complex and Characterization of the Regenerated Polymer

**DOI:** 10.3390/bioengineering7010028

**Published:** 2020-03-14

**Authors:** Diana Araújo, Vítor D. Alves, Ana C. Marques, Elvira Fortunato, Maria A. M. Reis, Filomena Freitas

**Affiliations:** 1 UCIBIO-REQUIMTE, Chemistry Department, Faculty of Science and Technology, NOVA University of Lisbon, 2829-516 Caparica, Portugal; df.araujo@campus.fct.unl.pt (D.A.); amr@fct.unl.pt (M.A.M.R.); 2 LEAF—Linking Landscape, Environment, Agriculture and Food, Instituto Superior de Agronomia, Universidade de Lisboa, Tapada da Ajuda, 1349-017 Lisbon, Portugal; vitoralves@isa.ulisboa.pt; 3 i3N/CENIMAT, Department of Materials Science, Faculty of Science and Technology, NOVA University of Lisbon and CEMOP/UNINOVA, Campus da Caparica, 2829-516 Caparica, Portugal; accm@campus.fct.unl.pt (A.C.M.); emf@fct.unl.pt (E.F.)

**Keywords:** chitin-glucan complex (CGC), chitosan-glucan complex (ChGC), NaOH/urea solvent systems, dissolution, structural analysis, thermal properties

## Abstract

Chitin-glucan complex (CGC) is a copolymer composed of chitin and glucan moieties extracted from the cell-walls of several yeasts and fungi. Despite its proven valuable properties, that include antibacterial, antioxidant and anticancer activity, the utilization of CGC in many applications is hindered by its insolubility in water and most solvents. In this study, NaOH/urea solvent systems were used for the first time for solubilization of CGC extracted from the yeast *Komagataella pastoris*. Different NaOH/urea ratios (6:8, 8:4 and 11:4 (w/w), respectively) were used to obtain aqueous solutions using a freeze/thaw procedure. There was an overall solubilization of 63–68%, with the highest solubilization rate obtained for the highest tested urea concentration (8 wt%). The regenerated polymer, obtained by dialysis of the alkali solutions followed by lyophilization, formed porous macrostructures characterized by a chemical composition similar to that of the starting co-polymer, although the acetylation degree decreased from 61.3% to 33.9–50.6%, indicating that chitin was converted into chitosan, yielding chitosan-glucan complex (ChGC). Consistent with this, there was a reduction of the crystallinity index and thermal degradation temperature. Given these results, this study reports a simple and green procedure to solubilize CGC and obtain aqueous ChGC solutions that can be processed as novel biomaterials.

## 1. Introduction

Chitin-glucan complex (CGC), a copolymer composed of chitin (N-acetyl-D-glucosamine polymer) and glucan (glucose homopolymer) moieties linked through β-(1,3) and β-(1,6) glycosidic linkages [1,2], is the main component of the inner cell wall of yeasts and fungi, contributing for the stiffness and stability of the cells [1,3]. Therefore, those microorganisms can be a source of CGC, whose biodegradability and biocompatibility, combined with its inherent bioactivity, render this biopolymer interesting for high-value applications. In fact, CGC of microbial origin has been reported to possess antibacterial [4], antioxidant [5] and anticancer activity [6], and has been used as an anti-aging component in cosmetics formulations [2], as a food additive [7], in wound dressings [8] and in pharmaceuticals for the treatment of obesity and diabetes [9].

Despite its proven properties, the utilization of CGC in a broader range of applications is still hindered by its insolubility in water and most organic and inorganic solvents [10,11]. Like chitin and cellulose, CGC solubility is limited to toxic and/or corrosive polar solvents (e.g., dimethylacetamide/LiCl) [12,13] and the resulting solutions are often unstable, which makes difficult their processing. CGC’s insolubility is caused by inter- and intramolecular hydrogen bonds established between CGC chains and the strong covalent linkages between β-glucan and chitin [14]. In order to improve CGC solubility, chemical modification (e.g., carboxymethylation) [15,16], and physical methods (e.g., sonication) that induce depolymerization [10], have been developed. However, those modifications may alter the physical-chemical properties of the original biopolymer, such as its biodegradability and bioactivity. Other strategies include the use of alternative solvents, such as ionic liquids (ILs), although some degree of CGC degradation was noticed as well [17].

In recent years, alkaline solvent systems, mainly based on NaOH or KOH, have been proposed for the dissolution of crustacean chitin [18,19], β-glucans [20,21] and cellulose [22,23] from plant sources, and yeast CGC [24]. The addition of urea to the alkali solution was reported to enhance the polymers’ solubilization by disrupting the inter- and intramolecular hydrogen bonds [25,26]. Moreover, it was reported that low temperature steps (below freezing point of the solvent system) plays an important role in the solubilization process. At such low temperatures, the hydrated alkali component disrupts the polymer chain matrix by breaking the hydrogen bonds and allowing the formation of new ones. This yields a stable structure composed of the polymer chain associated with the alkali component and water clusters [25,27]. The solubilization of the polymer chains is allowed by the volume expansion of the matrix upon freezing-induced strength and disruption of the hydrogen bonds.

In this study, NaOH/urea solvent systems were used for the first time for solubilization of CGC extracted from *Komagataella pastoris* biomass and the preparation of aqueous solutions, using a freeze/thaw procedure. Several concentrations of NaOH and urea were tested, and the rheological properties of the resulting solutions were studied. Moreover, the CGC was regenerated from such solutions and the resulting polymers were characterized in terms of acetylation degree, structural and thermal properties.

## 2. Materials and Methods

### 2.1. Materials

Yeast biomass was obtained by cultivation of *Komagataella pastoris* DSM 70877 using glycerol as the sole carbon source, as described by Farinha et al. [28]. CGC was extracted from *K. pastoris* biomass by the hot alkaline extraction procedure described by Araujo et al. [29], with slight modifications. Briefly, 1 L culture broth was mixed with 1 L NaOH 2 mol/L (to yield a suspension with a NaOH concentration of 1 mol/L) and treated at 65 °C for 2 h, under constant stirring. After centrifugation (13,000× g, 15 min), the alkaline-insoluble material was re-suspended in deionized water (200 mL), neutralized with H_2_SO_4_ 95% (Sigma-Aldrich, St. Louis, MO, USA) and washed repeatedly until constant conductivity values were reached (below 50 µS/cm), keeping a neutral pH. The CGC thus obtained was freeze dried (ScanVac CoolSafe^TM^, LaboGene, Lillerød, Denmark) at −110 °C for 48 h, and stored at room temperature in a closed vessel. NaOH pellets (99% purity) and urea (99% purity) were purchase from AzkoNobel (Amsterdam, the Netherlands) and Panreac (Barcelona, Spain), respectively.

### 2.2. CGC Dissolution in NaOH/Urea Solvent Systems

Three different NaOH/urea solvent systems were prepared with varying concentrations of each solute (Table 1). The selected NaOH/urea ratios were reported for solubilisation of crustacean chitin and/or cellulose [18,26,30,31,32]. For the dissolution experiments, the CGC powder (0.5 g) was dispersed in the solvent systems (25 g) and the suspensions were kept at −20 °C for 48 h. During this period, four freeze-thaw cycles were performed in which the thawed suspensions were extensively stirred (at 500 rpm, for 1 h), at room temperature. The insoluble fractions of the suspensions were separated from the soluble ones (first soluble fractions, coded as CGC_1.1_, CGC_2.1_ and CGC_3.1_, for solvent systems 1, 2 and 3, respectively) by centrifugation (20,000× g, 30 min, 4 °C). The obtained insoluble fraction was re-suspended in fresh NaOH/urea solvent system and subjected to the same freeze-thawing process to obtain a second soluble fraction (coded as CGC_1.2_, CGC_2.2_ and CGC_3.2_, for solvent systems 1, 2 and 3, respectively).

### 2.3. Polymer Regeneration

All soluble fractions were subjected to dialysis with a 12,000 MWCO membrane (Nadir^®^ dialysis tubing, Carl Roth, Karlsruhe, Germany) against deionized water, until neutral pH and constant conductivity values (20 µS/cm) were achieved. The samples were freeze dried and kept in closed vessels, at room temperature.

The overall polymer solubility (Sa) in each solvent system was determined by Equation (1):

Sa = ((W_1_ + W_2_)/W_initial_) × 100, (1)

where W_1_ is the weight (g) of first soluble fraction, W_2_ is the weight (g) of second soluble fraction and W_initial_ is the initial polymer weight (g). All experiments were performed in triplicate.

### 2.4. Rheological Properties of the Polymer Solutions

The rheological properties of solutions obtained with the three solvent systems were assessed using a controlled stress rheometer (HAAKE MARSIII, Thermo Scientific, Waltham, MA, USA) equipped with a cone-plate geometry (diameter 3.5 mm, angle 2°), with a gap of 0.1 mm. The samples were equilibrated at 20 °C, for 5 min, after which the flow curves were performed using a steady state flow ramp in the shear rate of 10–1000 s^−1^. The viscoelastic properties were evaluated by carrying out stress sweeps at a constant frequency (1 Hz) for a stress range from 10^−4^ to 1000 Pa; and frequency sweeps at a constant tension within the linear viscoelastic region, for a frequency range from 10^−3^ to 1 Hz.

### 2.5. Characterization of the Regenerated Polymer

#### 2.5.1. Elemental Analysis and Degree of Acetylation

Elemental analysis of the regenerated samples was performed by a Flash EA 1112 Series CHNS analyzer (Thermo Scientific). The chitin content (Q, %) was calculated based on the samples’ nitrogen content (%), using the following formula, Equation (2) [33]:

Q = 14.199 N,(2)

where N is the nitrogen content (%) in the sample.

The degree of acetylation (DA, %) was calculated by the following formula, Equation (3) [18]:

DA = ((C/N − 5.14)/1.72) × 100%,(3)

where C/N is the ratio (%) of carbon to nitrogen contents, as determined by elemental analysis.

#### 2.5.2. Fourier Transform Infrared Spectroscopy

Fourier transform infrared spectroscopy (FT-IR) was performed using a Nicolet 6700 FT-IR (Thermo Electron Corporation, Waltham, MA, USA) with a diamond crystal attenuated total reflectance (ATR) accessory.

#### 2.5.3. X-ray Diffraction Profiles

X-Ray Diffraction (XRD) was performed with a diffractometer (X’Pert Pro, PANalytical, Almelo, The Netherlands) with a CuKα target and wavelength of 1.5406 Å. The crystallinity index (CI, %) of the samples was determined by Equation (4) [28]:

CI (%) = ((I_110_ − I_am_)/I_110_) × 100, (4)

where I_110_ is the maximum intensity of the (110) peak at a 2θ angle around 19° and I_am_ is the intensity of the amorphous diffraction at 2θ ≈ 16°, corresponding to the minimum of intensity.

#### 2.5.4. Thermal Properties

Differential Scanning Calorimetry-Thermogravimetry analysis (DSC-TGA) was performed with a Simultaneous Thermal Analyser (STA 449 F3 Jupiter, NETZSCH Thermal Analysis, Wittelsbacherstraße, Germany), in an air atmosphere, with a heating rate of 20 °C/min, from 0 to 500 °C.

## 3. Results and Discussion

### 3.1. Preparation of CGC Solutions in NaOH/urea Solvent Systems

Previous studies reported the solubilization of yeast CGC, in alkali solutions (NaOH, KOH) for the preparation of hydrogels [24]. Moreover, it has been described that the addition of urea in aqueous alkali solutions improves the solubility and stability of cellulose [34] and crustacean chitin [18]. Therefore, in this study three solvent systems based on NaOH and urea at different ratios (6:8, 8:4 and 11:4 wt%, for solvent systems 1, 2 and 3, respectively) (Table 1) were prepared and tested to obtain CGC aqueous solutions using freeze/thaw procedures described for solubilisation of crustacean chitin [18,30,31] and cellulose [26,32].

**Figure 1:**
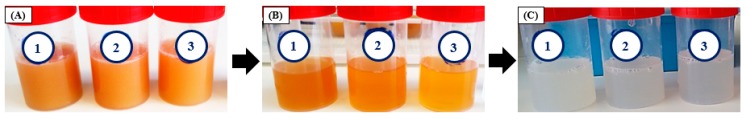
Macroscopic aspect of: (**A**) initial CGC suspensions in the three NaOH/urea solvent systems; (**B**) the CGC solutions obtained after removal of the insoluble fraction by centrifugation; (**C**) the corresponding CGC solutions after dialysis against deionized water. Solvent systems 1, 2 and 3 are represented by the numbers 1, 2 and 3, respectively.

CGC solubilization was observed for all tested solvent systems (Figure 1), although with slightly different solubility rates (Table 1). As shown in Figure 1, the obtained CGC solutions were opaque and presented a yellowish coloration (Figure 1A). After removal of the insoluble fraction, the solutions became translucid but kept the yellow color, which might be due to the presence of impurities (Figure 1B). During the dialysis process, impurities were removed and CGC solutions became white and opaque (Figure 1C).

The highest overall solubility (68.0 ± 1.7%) was obtained to solvent system 1 that contained the highest urea concentration (8 wt%) and the lowest NaOH concentration (6 wt%), suggesting that urea contributes for CGC solubilization. The same solvent system was reported to result in significantly lower solubility for shrimp shell chitin and cotton linter cellulose (30.0 and 36.1%, respectively) [18,26], even if only the first soluble fraction is considered (57.0 ± 1.4%).

The use of solvent systems 2 and 3, which had lower urea concentration (4 wt%) and higher NaOH concentrations (8 and 11 wt%, respectively), resulted in similar overall solubility values (65.2 ± 0.3 and 62.9 ± 3.1%, respectively) (Table 1). These results seem to indicate that increasing NaOH concentration in the solvent system, for the same urea concentration, has no significant impact on CGC solubilization. Hu et al. [18] and Zhou et al. [26] tested solvent system 2 for solubilization of shrimp shell chitin and cotton linter cellulose, respectively, achieving contrasting results. While a high solubility rate was reported for chitin in this solvent (85.0%) [18], a low solubility was obtained for cellulose (40.5%) [26].

These findings may be related to the differing nature of the macromolecules under study, namely, chitin, cellulose and CGC, which have distinct composition and molecular structures. Moreover, different procedures were adopted for chitin and cellulose solubilisation, namely, the initial polymer content, the number of freeze-thaw cycles and the exposure time to the solvent at low temperature, which have probably also influenced the reported results.

These results demonstrate that the tested NaOH/urea solvent systems were suitable to achieve high CGC solubilization, yielding solutions (first soluble fractions CGC_1.1_, CGC_2.1_ and CGC_3.1_) with polymer concentrations of 12.9 ± 1.1 wt% and 13.8 ± 0.2 wt% (Table 1). Furthermore, most of the polymer was solubilized during the first solubilization step since lower polymer concentrations were obtained in the second soluble fractions (below 3.4 ± 0.1 wt%).

The polymer concentration in the first soluble fractions for all tested solvent systems are within the range of those reported for crustacean chitin and cellulose using different types of solvents. In fact, similar polymer concentration (14%) was reported for the solubilization of chitin crab shell in dimethylacetamide/LiCl [35]. Lower values were also obtained using green solvents, such as deep eutectic solvents (DES) and ILs. Sharma et al. [36] reported the dissolution of crab chitin and cellulose in DES that resulted in concentration below 8 wt%. On the other hand, shrimp chitin solubilization in 1-ethyl-3-methyl-imidazolium bromide ([Emim][Br]) resulted in polymer concentrations of 12% [37].

### 3.2. Rheological Behavior of the Solutions

**Figure 2:**
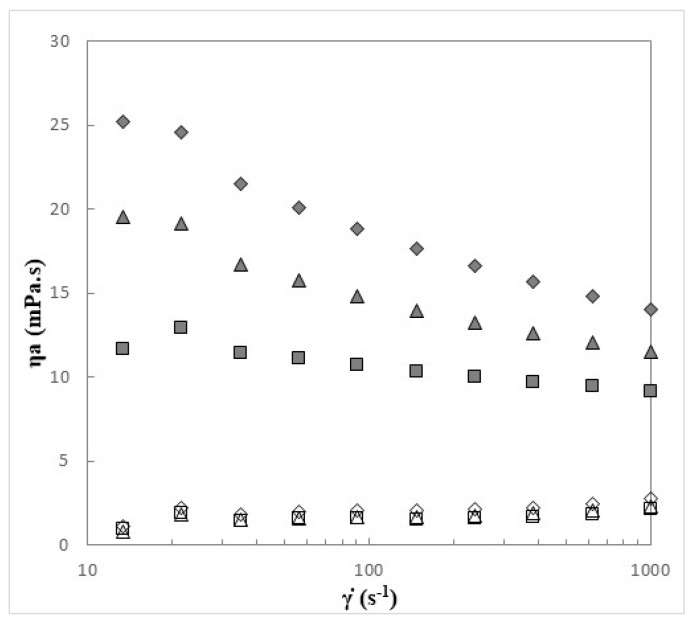
Apparent viscosity of first soluble fractions of CGC1 (■), CGC2 (▲) and CGC3 (◆), and solvent systems 1 (□), 2 (△) and 3 (◇), measured at 20 °C.

**Figure 3:**
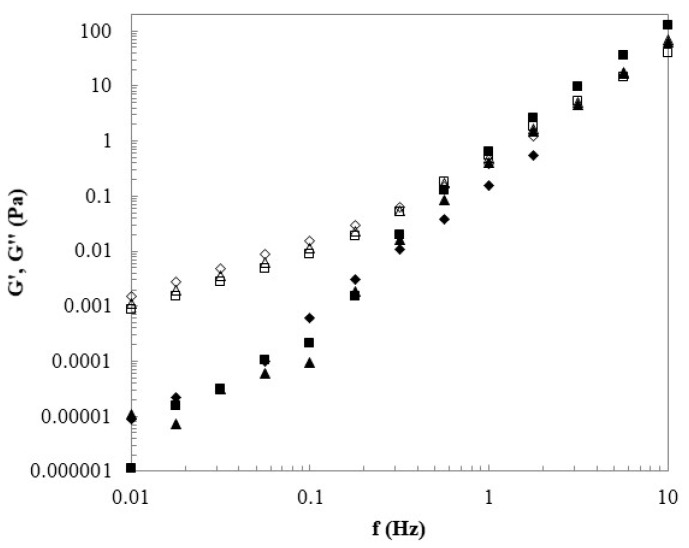
Viscoelastic properties of first soluble fractions of CGC1 (■), CGC2 (▲) and CGC3 (◆). Mechanical spectrum storage [G’ (full symbols)] and loss moduli [G’’ (open symbols)].

The rheological behavior of the first soluble fractions of CGC in each NaOH/urea solvent system (CGC_1.1_, CGC_2.1_ and CGC_3.1_) is shown in Figure 2 and Figure 3. The second soluble fraction was not analyzed due to their considerably low polymer concentrations.

The flow curves of the three solutions (Figure 2) had a similar behavior with a constant apparent viscosity at low shear rates (characteristics of Newtonian fluids), followed by a slightly decrease in apparent viscosity values with the increase of the shear rate (shear-thinning behavior). As shown in Figure 2, solution CGC_1.1_ approaches a Newtonian plateau at 11.6–12.9 mPa·s, while CGC_2.1_ and CGC_3.1_ solutions approached this plateau at higher values (19.1–19.5 and 24.6–25.2 mPa·s, respectively).

Similar flow behavior was reported by Hu et al. [18] for shrimp chitin solutions in NaOH/urea at a concentration of 2 wt%. In that study, the solutions were reported to exhibit a Newtonian plateau at low shear rates (0.1–1.0 s^−1^), followed by a shear-thinning behavior with the increase of shear rate [19]. The observed flow behavior of the polymer solutions was not due to the solvent systems that exhibited a quite low viscosity (below 2.2 mPa.s) and no shear thinning behavior (Figure 2).

Figure 2 shows that solution CGC_1.1_ presented the lowest apparent viscosity values (11.7 mPa·s, at 13.3 s^−1^), while CGC_2.1_ and CGC_3.1_ had slightly higher values (19.5 and 25.2 mPa·s at 13.3 s^−1^, respectively). Since the polymer concentration was similar for all three solutions (12.91–13.79 wt%), these results might suggest that the flow behavior was influenced by the solvent systems’ composition. The low concentration of NaOH (6 wt%) and high urea content (8 wt%) present in solvent 1 might have prevented the interaction of the polymer macromolecules, thus decreasing the viscosity of solution CGC_1.1_. Similar behavior was reported by Huber et al. [38] for cellulose solutions in NaOH/urea, in which a viscosity reduction of almost 40% was observed by increasing the urea concentration in the solvent.

On the other hand, increasing the concentration of NaOH in solvent systems 2 and 3 (to 8 and 11 wt%, respectively) has apparently led to higher apparent viscosity in solutions CGC_2.1_ and CGC_3.1_ (Figure 2). The increasing ionic strength of these solutions may have contributed to the higher apparent viscosity observed.

The mechanical spectra (Figure 3) revealed that all the solutions presented a loss modulus (G’’) considerably higher than the storage modulus (G’), both highly dependent on the frequency. This result is indicative of liquid-like fluids behavior, since the polymer solutions show a higher viscous behavior than the elastic one. As the frequency increases, both moduli tend to the same value. A similar trend was reported for chitin in NaOH/urea aqueous solution. Hu et al. [39] demonstrated that chitin solutions with concentration range from 0.5 to 1.5% also present values of G’’ higher than G’ for low frequency values. In the case of chitin solutions, at low concentrations (0.5 and 1%) both moduli have different values at frequency 1 Hz which is consistent with a dilute solution. Once concentration is increased to 1.5%, analogous with the results obtained for CGC solutions, a cross-over is observed at a frequency below 1 Hz.

### 3.3. Characterization of the Regenerated Polymer

#### 3.3.1. Elemental Analysis and Degree of Acetylation

The polymers regenerated from the soluble fractions in solvent systems 1 (CGC_1.1_ and CGC_1.2_), 2 (CGC_2.1_ and CGC_2.2_) and 3 (CGC_3.1_ and CGC_3.2_) were characterized in terms of their physical and chemical composition. Table 2 shows the chemical characterization of the original CGC (CGC_0_) and the polymers regenerated from all solutions.

The chitin content of the original CGC_0_, as determined based on the elemental analysis data, was 23.8 ± 0.10%. The first soluble fractions (CGC_1.1_, CGC_2.1_ and CGC_3.1_) had lower chitin contents (18.8 ± 2.11, 20.7 ± 0.40 and 23.4 ± 2.61%, respectively, Table 2). Interestingly, the second soluble fractions (CGC_1.2_, CGC_2.2_ and CGC_3.2_) were apparently richer in chitin, with higher content values (21.7 ± 0.40, 26.5 ± 2.31 and 32.8 ± 1.61%, respectively) than the first soluble ones. This might be explained by the fact that the CGC_0_ sample was probably composed of polymer chains of different composition in terms of chitin:glucan ratio, which may have been solubilized differently during the freeze/thaw procedure. It is likely that the macromolecule chains with a lower chitin:glucan ratio were more easily solubilized, thus resulting in solutions with lower chitin content (i.e., the first soluble fractions CGC_1.1_, CGC_2.1_ and CGC_3.1_), while the chains with higher chitin:glucan ratio only solubilized later, during the second solubilization step (i.e., the second soluble fractions CGC_1.2_, CGC_2.2_ and CGC_3.2_).

On the other hand, it can be also observed that the chitin content of the regenerated polymers increased as the NaOH concentration in the solvent systems increased (Table 2). In fact, as the NaOH concentration increased (6, 8 and 11%, in solvents 1, 2 and 3, respectively), the chitin content of the corresponding regenerated polymers also increased (18.8 ± 2.11, 20.7 ± 0.40 and 23.4 ± 2.61% for the first soluble fractions, and 21.7 ± 0.40, 26.5 ± 2.31 and 32.8 ± 1.61% for the second soluble fractions). These results are in accordance with the study reported by Hu et al. [18], in which the solubilization of chitin was improved from 30% to 85% by increasing the NaOH concentration by 2% in the NaOH/urea solvent system.

The dissolution process had a significant impact on the chitin acetylation degree. Initially, CGC_0_ presented a DA of 61.3 ± 0.41%, while the regenerated polymers presented lower values of acetylation degree (between 33.9 ± 0.99 and 50.6 ± 0.99%) indicating that chitin was converted into chitosan, its soluble and N-deacetylated derivative [40,41]. Focusing on the regenerated polymers, concomitant with the values obtained for chitin content, the second soluble fractions display higher degree of acetylation when comparing with the first ones (Table 2). According to these results, it can be suggested that chitin molecules with higher concentration of N-acetyl-glucosamine monomers on their structure are more difficult to solubilise and it was only possible after the second dissolution phase.

#### 3.3.2. Fourier Transform Infrared Spectroscopy

**Figure 4:**
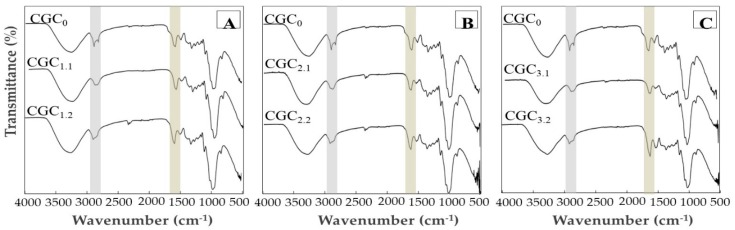
FTIR spectra of CGC_0_ and the polymer regenerated from the soluble fractions of CGC in: (**A**) solvent system 1 (CGC_1.1_ and CGC_1.2_), (**B**) solvent system 2 (CGC_2.1_ and CGC_2.2_) and (**C**) solvent system 3 (CGC_3.1_ and CGC_3.2_).

The FTIR spectra of the CGC_0_ and the co-polymers regenerated from the NaOH/urea solvent systems are presented in Figure 4. Despite the structural differences related with the chitin deacetylation, no significant impact on the chemical structure was noticed. All spectra presented a broad and intense band around 3400 cm^−1^ (Figure 4), characteristic of O–H stretching of hydroxyl groups, which is common to chitin/chitosan and glucan polymers [16,42,43]. As reported by Farinha et al. [28], this band overlaps the N–H (asymmetric) and N–H (symmetric) stretching peaks. Two peaks, corresponding to the C–H stretching of CH_3_ and CH_2_ groups, appear at 2916 and 2848 cm^−1^, respectively. For CGC_0_, the intensity of those peaks is higher, when comparing with the spectra of the regenerated polymers CGC1, CGC2 and CGC3. This difference was expected since these peaks are more evident in chitin than chitosan [44]. Additionally, it can be noticed that a decrease in the absorption at around 1650 cm^−1^ occurs for the regenerated polymers, which is associated with the frequency of the vibration modes of amide I (Figure 4). This reduction in intensity is higher for the first soluble fractions, which is concomitant with the lower chitin content of CGC_1.1_, CGC_2.1_ and CGC_3.1_ polymers (Table 2). Similarly, the N–H deformation of amide II and C–N stretching in amide III (1550 and 1311 cm^−1^, respectively) [45] which are present in all the spectra but with low intensity in the CGC_1.1_, CGC_2.1_ and CGC_3.1_.

The nature of the linkages between β-glucan units can also be assessed by FTIR spectroscopy [43]. As described by Farinha et al. [28], β-1,3-glucan linkages are represented by small peaks at 890, 1156 and 1370 cm^−1^ and characteristic peaks of β-1,6-glucans linkage are noticed around 920, 1045 and 1730 cm^−1^. Figure 4 shows that all the polymers presented the small peaks assigned to β-1,3-glucans linkages. However, due to the low content of β-1,6-glucans in *K. pastoris* CGC, the peaks attributed to this linkage are vague and doubtful in all the spectra, including in CGC_0_.

#### 3.3.3. XRD Analysis

**Figure 5:**
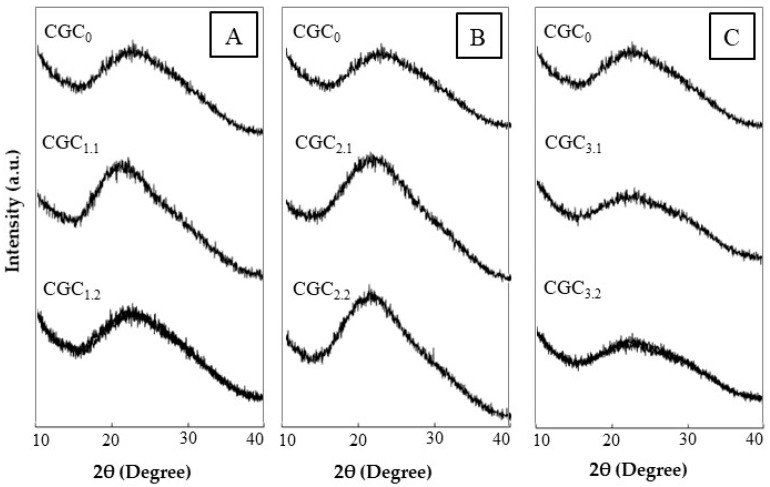
X-ray diffraction profiles of CGC_0_ and the polymer regenerated from the soluble fractions of CGC in: (**A**) solvent systems 1 (CGC_1.1_ and CGC_1.2_), (**B**) solvent systems 2 (CGC_2.1_ and CGC_2.2_) and (**C**) solvent systems 3 (CGC_3.1_ and CGC_3.2_).

Similar XRD patterns were obtained for CGC_0_ and the regenerated polymers (Figure 5). The diffractograms of all the polymers presented a broad peak around 2θ ~ 20°, which is characteristic of amorphous polymers [46]. These results are in accordance with Farinha et al. [28] that reported a large peak at this region for *K. pastoris* CGC. The authors described the amorphous nature of chitin and glucan co-polymers mainly due to the presence of high β-glucan contents.

Nevertheless, the peak intensity was different for CGC_0_ and the regenerated polymers, reflecting differences in the polymers’ crystallinity. The CI of CGC_0_ determined based on the XRD data was 35%. This value is lower than the one reported in the literature for CGC from *K. pastoris* (50%) composed of 24.6 mol% of chitin [28]. Despite this difference, the low crystallinity value was expected due to the presence of β-glucans within the co-polymer’s structure. Considering the regenerated polymers, apparently the exposure to the NaOH/urea solvent systems and the dissolution procedure, led to a decrease in the CI to 23–32% (Table 3).

These results may be related with the DA of the polymers. As suggested by Kumirska et al. [47], lower DA values lead to a decrease of the intensity of the peak and, consequently, to a decrease in crystallinity. Additionally, Seoudi and Nada [48] reported that CI of chitin crab shell decreased after treatment with NaOH, which caused hydrolysis of acetamide groups. Similar results were obtained for cellulose, where a decrease of around 8% in CI was verified after the dissolution with NaOH/urea aqueous solution [49].

#### 3.3.4. Thermal Properties

The TGA demonstrates that CGC_0_ and the regenerated polymers exhibited similar profiles, comprising three of thermal degradation steps. The main degradation (Δm ≈ 60%) proceeds in the second step that occurs between 220 and 320 °C, corresponding to the degradation of saccharide structure including dehydration of the saccharide rings, depolymerisation of the branched part of the molecules and decomposition of acetylated and deacetylated units of chitin [28]. The third degradation step occurring at temperatures above 320 °C might be related to the destruction of pyranose rings.

**Figure 6:**
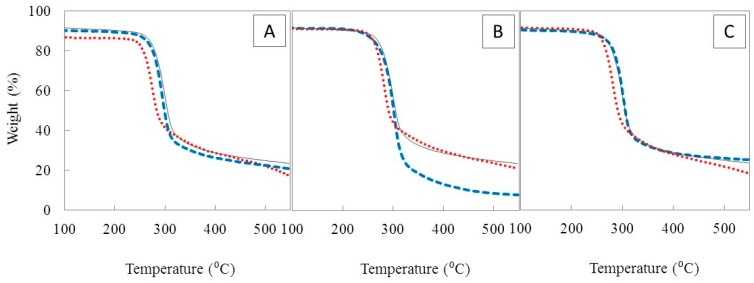
Thermogravimetric analysis (TGA) of the polymers: CGC1 (**A**), CGC2 (**B**) and CGC3 (**C**). Continuous line represents CGC_0_; Dotted and dashed lines corresponds to first and second soluble fractions, respectively.

For CGC_0_, the degradation temperature (T_deg_) was 302 °C and there was a char yield of 26%, at 550 °C (Figure 6). The results are in accordance with the ones reported by Farinha et al. [28] where the T_deg_ of CGC from *K. pastoris* was found to be 315 °C.

The polymers regenerated from the first soluble fractions (CGC_1.1_, CGC_2.1_ and CGC_3.1_) had lower T_deg_ (250 °C, 256 °C and 267 °C, respectively). This result is probably due to the lower DA (33.9–41.6%) and CI (23–32%) of those polymers compared to CGC_0_ (DA = 61.3% and CI = 35%). On the other hand, the polymers regenerated from the second soluble fractions (CGC_1.2_, CGC_2.2_ and CGC_3.2_), owing to the higher DA (44.8–50.6%) and CI (25–32%), had higher T_deg_ (293–302 °C) similar to CGC_0_ (302 °C). As expected, these results revealed the lower thermal stability of the regenerated polymers is a consequence of the decreased acetyl content since chitosan molecules are known for their lower thermal stability compared to chitin [50].

## 4. Conclusions

Three different water based solvent systems were designed to dissolve CGC, a biopolymer known for its intractability due to insolubility in water and most organic solvents. High polymer solubility (over 60%) was achieved for all the tested solvent systems. The solutions rheological properties showed similar shear thinning behavior with Newtonian plateaus at shear rates between 13.3 and 21.5 s^−1^, and liquid-like fluid behavior for all solutions. The characterization of the regenerated polymers obtained by freeze drying the solutions indicated that the original CGC co-polymer was deacetylated, yielding chitosan-glucan complex (ChGC) that was characterized by lower crystallinity and lower T_deg_. This study demonstrated the feasibility of using a water based green solvent to dissolve CGC, thus opening up for the possibility of processing this biopolymer in different areas of application as a novel biomaterial.

## Figures and Tables

**Table 1:** Solubilized CGC mass, polymer concentration and overall solubilization of the soluble fractions in different NaOH/urea solvent systems.

Solvent System	NaOH:Urea (wt%:wt%)	Starting Polymer Mass (mg)	Fraction Solution	Solubilized Polymer Mass (mg)	Polymer Concentration (wt%)	Solubility (%)	Overall Solubility (%)
1	6:8	504.4 ± 5.1	CGC_1.1_ CGC_1.2_	275.8 ± 4.1 67.2 ± 0.9	13.8 ± 0.2 3.4 ± 0.1	54.7 ± 1.4 13.3 ± 0.3	68.0 ± 1.7
2	8:4	506.0 ± 4.7	CGC_2.1_ CGC_2.2_	275.4 ± 6.0 54.4 ± 10.8	13.8 ± 0.3 2.7 ± 0.5	54.4 ± 1.7 10.7 ± 2.0	65.2 ± 0.3
3	11:4	502.7 ± 1.2	CGC_3.1_ CGC_3.2_	258.3 ± 22.3 57.8 ± 7.5	12.9 ± 1.1 2.9 ± 0.4	51.4 ± 4.6 11.5 ± 1.5	62.9 ± 3.1

**Table 2:** Chemical characterization of the original CGC (CGC_0_) and polymer samples regenerated from the NaOH/urea solvent systems (DA, degree of acetylation).

Sample	Elemental Analysis (%)			Chitin Content (%)	DA (%)
	C	H	N		
CGC_0_	43.6 ± 0.05	7.2 ± 0.05	1.7 ± 0.01	23.8 ± 0.10	61.3 ± 0.41
CGC_1.1_	40.4 ± 0.17	7.2 ± 0.05	1.3 ± 0.15	18.8 ± 2.11	34.5 ± 1.40
CGC_1.2_	42.0 ± 0.09	7.2 ± 0.10	1.5 ± 0.03	21.7 ± 0.40	47.6 ± 0.76
CGC_2.1_	40.3 ± 0.12	7.2 ± 0.09	1.5 ± 0.03	20.7 ± 0.40	33.9 ± 0.99
CGC_2.2_	41.6 ± 0.35	7.0 ± 0.18	1.9 ± 0.16	26.5 ± 2.31	44.8 ± 2.86
CGC_3.1_	41.2 ± 0.22	7.1 ± 0.06	1.7 ± 0.18	23.4 ± 2.61	41.6 ± 1.81
CGC_3.2_	42.3 ± 0.12	6.9 ± 0.06	2.3 ± 0.11	32.8 ± 1.61	50.6 ± 0.99

**Table 3:** Degradation temperature (Tdeg) and crystallinity index (CI) of the original. CGC (CGC_0_) and polymer samples regenerated from the NaOH/urea solvent systems.

Sample	CGC_0_	CGC_1.1_	CGC_1.2_	CGC_2.1_	CGC_2.2_	CGC_3.1_	CGC_3.2_
T_deg_ (°C)	302	250	293	256	300	267	302
CI (%)	35	28	30	32	32	23	25
